# Expansion of myeloid‐derived suppressor cells with aging in the bone marrow of mice through a NF‐κB‐dependent mechanism

**DOI:** 10.1111/acel.12571

**Published:** 2017-02-23

**Authors:** Rafael R. Flores, Cheryl L. Clauson, Joonseok Cho, Byeong‐Chel Lee, Sara J. McGowan, Darren J. Baker, Laura J. Niedernhofer, Paul D. Robbins

**Affiliations:** ^1^ Department of Metabolism and AgingThe Scripps Research Institute‐FloridaJupiterFL33458USA; ^2^ Molecular Genetic and MicrobiologyHillman Cancer CenterUniversity of Pittsburgh Cancer InstitutePittsburghPA15213USA; ^3^ Division of Hematology and OncologyDepartment of MedicineUniversity of Pittsburgh School of MedicinePittsburghPA15232USA; ^4^ Department of Pediatric and Adolescent MedicineMayo Clinic College of MedicineRochesterMN55905USA

**Keywords:** myeloid‐derived suppressor cell, NF‐κB, senescence

## Abstract

With aging, there is progressive loss of tissue homeostasis and functional reserve, leading to an impaired response to stress and an increased risk of morbidity and mortality. A key mediator of the cellular response to damage and stress is the transcription factor NF‐κB. We demonstrated previously that NF‐κB transcriptional activity is upregulated in tissues from both natural aged mice and in a mouse model of a human progeroid syndrome caused by defective repair of DNA damage (ERCC1‐deficient mice). We also demonstrated that genetic reduction in the level of the NF‐κB subunit p65(RelA) in the *Ercc1* ^−/∆^ progeroid mouse model of accelerated aging delayed the onset of age‐related pathology including muscle wasting, osteoporosis, and intervertebral disk degeneration. Here, we report that the largest fraction of NF‐κB ‐expressing cells in the bone marrow (BM) of aged (>2 year old) mice (C57BL/6‐NF‐κB^EGFP^ reporter mice) are Gr‐1^+^CD11b^+^myeloid‐derived suppressor cells (MDSCs). There was a significant increase in the overall percentage of MDSC present in the BM of aged animals compared with young, a trend also observed in the spleen. However, the function of these cells appears not to be compromised in aged mice. A similar increase of MDSC was observed in BM of progeroid *Ercc1* ^−/∆^ and *BubR1* ^H/H^ mice. The increase in MDSC in *Ercc1* ^−/∆^ mice was abrogated by heterozygosity in the p65/RelA subunit of NF‐κB. These results suggest that NF‐κB activation with aging, at least in part, drives an increase in the percentage of MDSCs, a cell type able to suppress immune cell responses.

## Introduction

Aging is characterized by a loss of tissue homeostasis and an impaired ability to respond to stress, which results in a dramatically increased risk of morbidity and mortality (Rattan, 2006; van Deursen, 2014). On a molecular level, DNA damage, mitochondrial dysfunction, telomere shortening, and the accumulation of macromolecular waste are all thought to contribute to driving aging. This cellular damage promotes aging by leading to dysfunction, apoptosis, or cellular senescence (Rattan, 2006; van Deursen, 2014).

A key mediator of the cellular response to damage and stress is the heterodimeric transcription factor NF‐κB (Gasparini & Feldmann, 2012). The NF‐κB cascade canonically organizes and executes pro‐inflammatory transcriptional programs. The NF‐κB family consists of five transcription factors p50, p52, p65, c‐REL, and RelB (regulated by IκB proteins). In unstimulated cells, NF‐κB is associated with IκB proteins and is sequestered in the cytoplasm. When stimulated, IκB is phosphorylated by IκB kinase, ubiquitinized, and degraded in the proteasome. NF‐κB is then released and translocated into the nucleus, where it can activate promoters. The IKK complex is composed of the catalytic subunits IKKα and IKKβ, and a regulatory subunit IKKγ (NEMO). IKKβ coordinates the classical arms of the two distinct NF‐kB activation pathways and mediates the major activity of IKK. In particular, genotoxic and oxidative stress triggers the activation of NF‐κB through the ATM (ataxia telangiectasia mutated kinase), which, in turn, can induce cellular senescence and inflammation (Tilstra *et al*., 2011; McCool & Miyamoto, 2012). Following activation by DNA damage, ATM associates with NEMO, a component of the IKK regulatory complex. The association of ATM with NEMO leads to the phosphorylation of NEMO, resulting in the shuttling of this complex from the nucleus to the cytoplasm where it binds and activates the IKK complex (IKKα and IKKβ). IKK phosphorylates IκB, resulting in its degradation and permitting nuclear translocation and thus activation of NF‐κB (Oeckinghaus *et al*., 2011; Ruland, 2011).

We demonstrated previously that NF‐κB transcriptional activity is upregulated stoichastically in a variety of tissues with both natural aging and in a mouse model of a human progeroid syndrome caused by defective repair of DNA damage, *Ercc1* ^−/∆^ hypomorphic mouse (Tilstra *et al*., 2012). In addition, genetic reduction in the level of the NF‐κB subunit p65/RelA in *Ercc1* ^−/∆^ mice, with a lifespan 6 months, delayed the onset of age‐related pathology including osteoporosis, neurodegeneration, BM hypoplasia, epidermal atrophy, sarcopenia, liver and kidney dysfunction. Similarly, inhibition of NF‐κB activation by chronic, systemic administration of a peptide inhibitor of the IKK complex, 8K‐NBD, altered gene expression networks, improved pathology, and delayed the onset of age‐related disease in the *Ercc1* ^−/∆^ mice.

Myeloid‐derived suppressor cells (MDSCs) represent a subset of myeloid cells that originate in the BM with not fully understood actions (Gabrilovich & Nagaraj, 2009; Ribechini *et al*., 2010; Talmadge & Gabrilovich, 2013). MDSC suppression on lymphocyte function can occur through cytokines such as IL‐10, through direct receptor–ligand interactions, and via short‐lived chemical mediators (Gabrilovich & Nagaraj, 2009; Talmadge & Gabrilovich, 2013). MDSCs also can suppress T‐cell function through a mechanism involving the production of reactive oxygen species (ROS) (Corzo *et al*., 2009; Lu *et al*., 2011). These mechanisms collectively increase the threshold of activation for lymphocytes, thereby preventing inappropriate responses to self‐proteins.

Several studies have suggested there is an increase in the frequency of MDSCs in both elderly patients and aged mice (Hurez *et al*., 2012; Jackaman *et al*., 2013; Verschoor *et al*., 2013). In naturally aged mice, the increase in the percent of MDSCs was observed in the spleen, lymph nodes, and BM (Enioutina *et al*., 2011; Hurez *et al*., 2012; Jackaman *et al*., 2013). However, most of the studies have been performed during cancer development where the percentage of MDSC is much higher than in ‘healthy age’ conditions.

The overall aim of this study was to understand in what cell types NF‐κB becomes activated with age and how this activation affects the aging process. Using mice transgenic for an EGFP reporter under the regulation of a NF‐κB ‐dependent promoter (NF‐κB^EGFP^), we observed an increase in the percent of EGFP‐positive cells in different tissues with natural aging (Tilstra *et al*., 2012; unpublished observations). Here we demonstrate that the EGFP^+^ cells found in the BM of naturally aged mice are positive for CD11b^+^ and Gr‐1^+^, markers used to identify MDSCs. We also observed a significant increase in the overall fraction of MDSCs, both EGFP^+^ and EGFP^−^, in the BM. A similar increase in Gr‐1^+^CD11b^+^ cells was observed in the BM of two different progeroid mouse models, *Ercc1* ^−/∆^ and BubR1^H/H^. Heterozygosity in the p65/RelA subunit of NF‐κB in the *Ercc1* ^−/∆^ mice prevented the increase in the Gr‐1^+^CD11b^+^. These results suggest that both natural and accelerated aging drives an expansion in MDSC, a cell type known to suppress both the adaptive and innate immune response, in part through a NF‐κB‐dependent mechanism.

## Results

### Expansion of NF‐κB^EGFP+^Gr‐1^+^CD11b^+^ cells in the BM of natural aged mice

**Figure 1:**
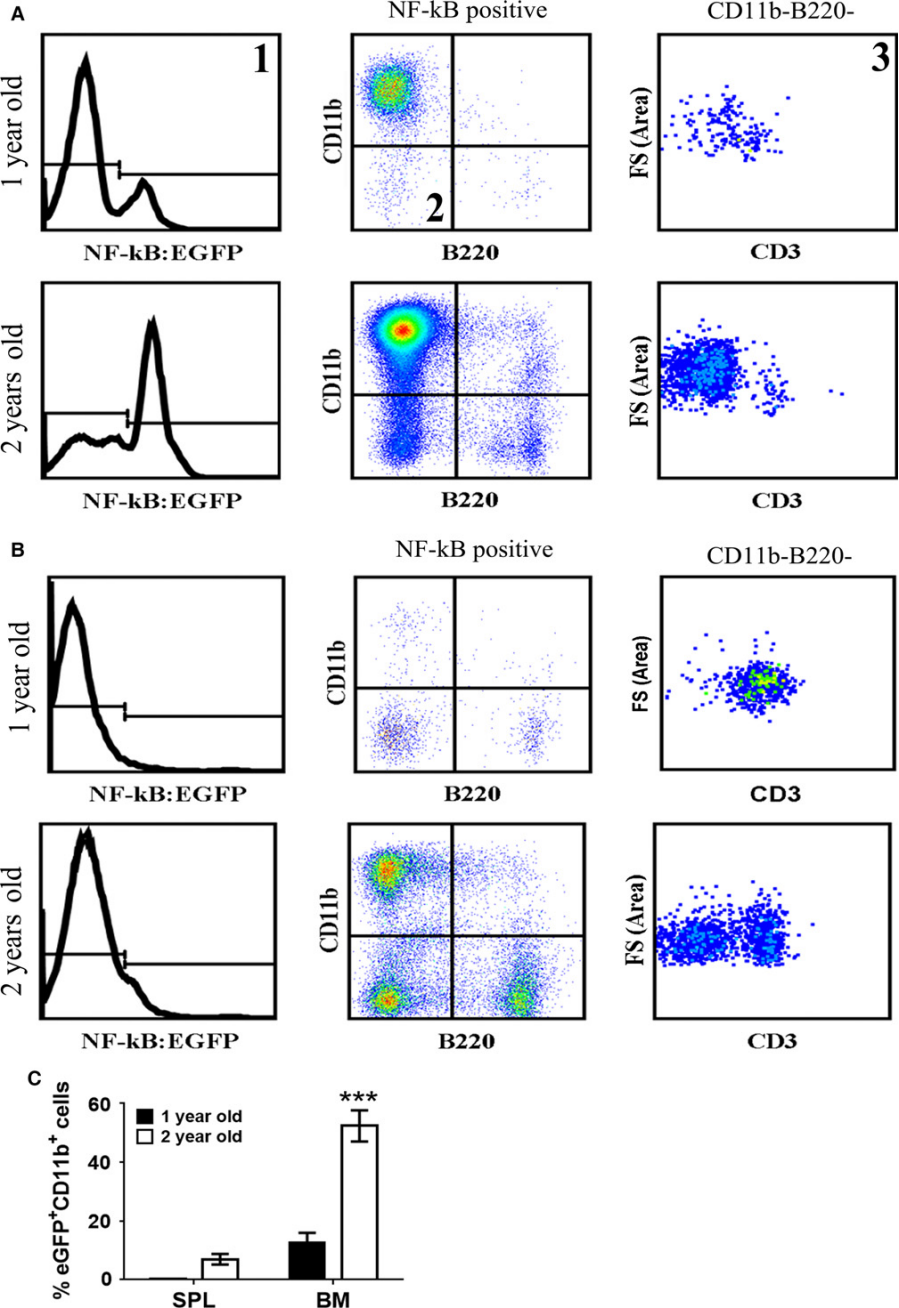
NF‐κB is highly activated in CD11b^+^ cells of BM with age. BM was extracted from the femur and tibia of the hind legs of mice. RBCs were depleted and the BM was washed extensively prior to flow cytometry. As shown in histogram (1), NF‐κB^EGFP^‐positive cells were selected. From the NF‐κB^EGFP+^ cells, CD11b^+^ cells were gated against B220^+^ cells (dot plot 2). The majority of the NF‐κB^EGFP+^ cells were CD11b^+^ in the BM (A) and in spleen (B). The remaining NF‐κB^EGFP^‐positive cells (i.e., CD11b B220 double negative cells) were CD3^+^ T cells in the BM (A) (dot plot 3) and particularly in the spleen (B). (C) The percentage of EGFP^+^CD11b^+^ cells in the BM and SPL of naturally aged mice is shown. The results shown represent three young mice and three old mice. The data were analyzed using nonparametric two‐way ANOVA (Mann–Whitney) ± SEM (***P < 0.001).

To assess the impact of NF‐κB on the aging process, we utilized C57BL/6 mice transgenic for a synthetic NF‐κB‐dependent promoter driving an EGFP reporter (NF‐κB^EGFP^) (Magness *et al*., 2004). We detected an increase in the number of EGFP‐positive cells in old mice (≥ 2 years) in several tissues including the gastrointestinal tract, lungs, and pancreas (Tilstra *et al*., 2012; data not shown). In these mice, the percent of EGFP^+^ cells was particularly high in the BM (Fig. 1A, histogram 1). In the BM, the majority of NF‐kB^EGFP+^ cells were also CD11b positive (dot plot 2), which was greater in 2‐year‐old mice than in 1‐year‐old mice (Fig. 1A). T and B cells constituted the rest of the age‐dependent NF‐κB^EGFP^ activity in the BM (dot plot 3). In contrast, there was a marginal increase in the percent of EGFP‐positive cells in the spleen of 2‐year‐old mice (Fig. 1B) with a distribution similar to the BM. Quantitative analysis showed that there was a significant increase in the percentage of EGFP^+^CD11b^+^ cells in both the SPL and BM (Fig. 1C).

**Figure 2:**
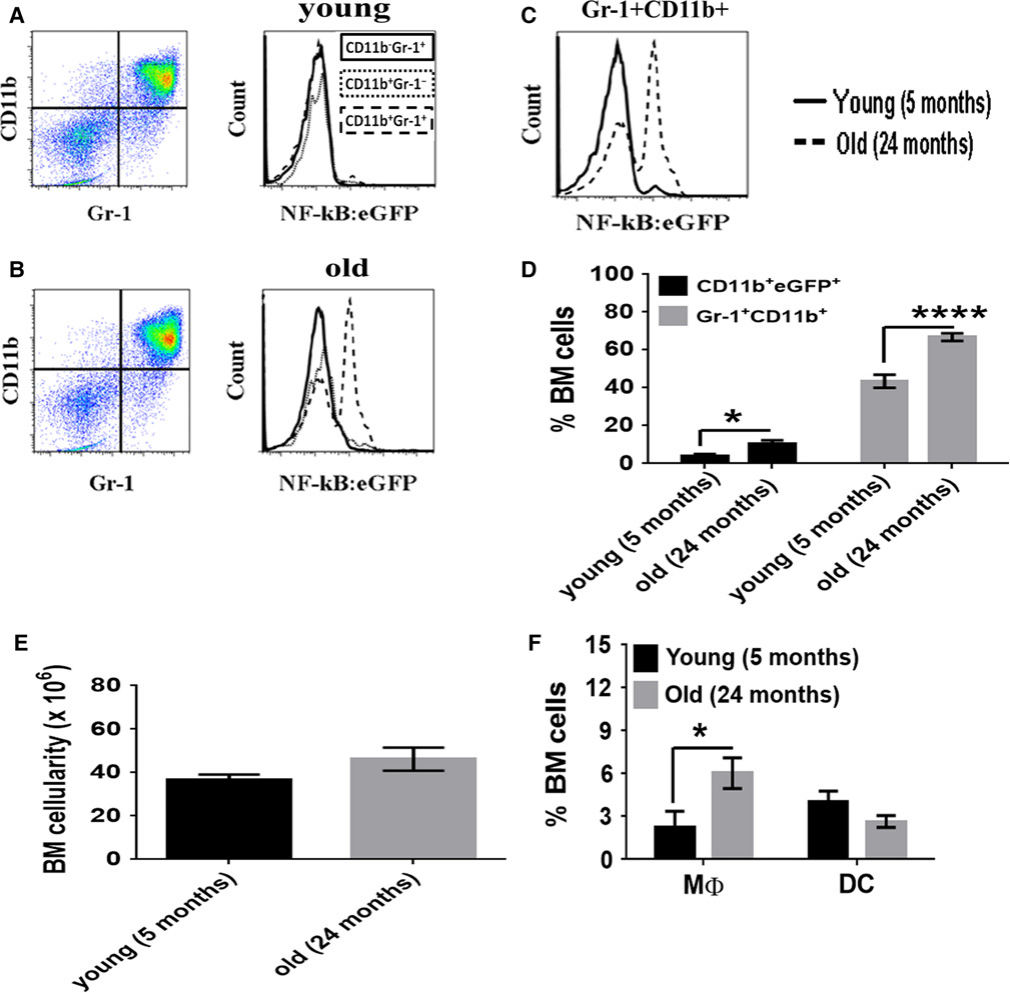
Expansion of NF‐κB^EGFP^ myeloid suppressor cells in the BM of naturally aged mice. BM was extracted from the femur and tibia of the hind legs of mice. RBCs were depleted and the BM was washed extensively prior to flow cytometry. Dot plots of Gr‐1^+^CD11b^+^ cells from young (A) and old (B) mice are shown. The expression of EGFP in Gr‐1^+^CD11b^+^ cells (dash) compared with Gr‐1 (solid) and CD11b (dotted) single positive cells is shown in adjacent histograms. (C) The expression of EGFP by Gr‐1^+^CD11b^+^ cells from old and young mice is directly compared. (D) Quantitative analysis comparing the frequency of CD11b^+^EGFP^+^ and Gr‐1^+^CD11b^+^ between old and young mice. (E) The cellularity of the BM is shown. (F) The percent of MΦ (CD11b^+^F4/80^+^) and DC (CD11b^+^CD11c^+^) in the BM is shown. The results shown represent four young mice and eight aged mice. The data were analyzed using nonparametric two‐way ANOVA (Mann–Whitney) ± SEM (*P < 0.05, ****P < 0.0001).

We focused our attention on identifying cells that expressed high levels of EGFP in addition to CD11b; thus, we probed the BM for the expression of innate immune cell markers Gr‐1, CD11c, and F4/80 (Fig. 2). A majority of the EGFP^+^ signal present in the BM of old mice was detected in Gr‐1^+^CD11b^+^ cells, which in young mice was expressed at substantially lower levels in this population of cells (Fig. 2A and B). Both Gr‐1 and CD11b are markers for MDSCs, which, as previously stated, represents a heterogeneous group of immune cells encompassing cells from myeloid progenitors to immature differentiated myeloid cells (Gabrilovich & Nagaraj, 2009; Ribechini *et al*., 2010; Talmadge & Gabrilovich, 2013). In the BM, a majority of the EGFP^+^ cells were Gr‐1^+^CD11b^+^ MDSC (Fig. S1). However, in the spleen, most of the EGFP^+^ cells were either MDSCs (Fig. S1) or CD11b single positive cells (data not shown). Further analysis revealed that the expression of EGFP by Gr‐1^+^CD11b^+^ in the BM of old mice was considerably higher in these cells than cells that were either single positive for CD11b (dotted histogram) or Gr‐1 (solid histogram) which in young mice turned out to be much less (Fig. 2A,B). A direct comparison between the two age groups revealed that EGFP was expressed at higher levels in MDSC from old mice than young mice (Fig. 2C). There was also a greater percentage of MDSCs present in the BM of old mice (Fig. 2D), including both EGFP‐positive and EGFP‐negative MDSCs, than in young mice. However, we observed no difference in the cellularity of the BM between old and young mice (Fig. 2E). Additionally, we detect no differences in the distribution of MDSC subsets in the BM of old or young mice (CD11b^+^Ly‐6G^hi^Ly‐6C^lo^ granulocytic MDSC and CD11b^+^Ly‐6C^+^Ly‐6G^lo^ monocytic MDSC; Fig. S2). However, we did detect a significantly greater percentage of CD11b^+^F4/80^+^ macrophages (MΦ) present in the BM of old mice but not that of CD11b^+^CD11c^+^ dendritic cells (DC) (Fig. 2F). Analysis of MΦ and DCs from the BM of old mice exhibited higher levels of NF‐κB^EGFP^ in these cells than in young mice (Fig. S3). Independent of the age of the mouse, NF‐kB^EGFP+^ MΦ and DC expressed higher levels of CD86 (Fig. S4).

**Figure 3:**
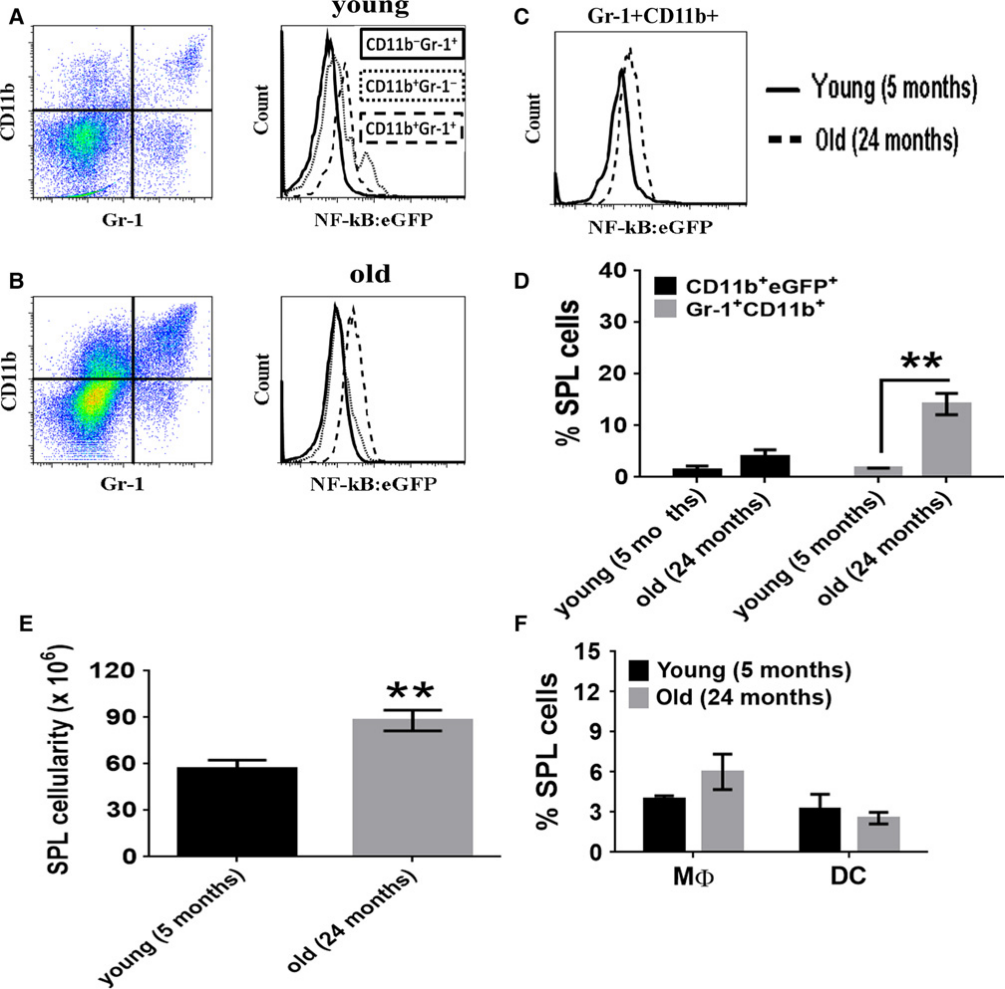
Significant expansion of NF‐κB^EGFP^ myeloid suppressor cells in the spleens of naturally aged mice. Splenocytes were depleted of RBC and washed extensively prior to flow cytometry. Gr‐1^+^CD11b^+^ cells from young (A) and old (B) mice are shown in dot plots. The expression of EGFP in Gr‐1^+^CD11b^+^ cells (dash) was compared to Gr‐1 (solid) and CD11b (dotted) single positive cells and shown in adjacent histograms. (C) The expression of EGFP by Gr‐1^+^CD11b^+^ cells from old and young mice is directly compared. (D) Quantitative analysis comparing the frequency of CD11b^+^EGFP^+^ and Gr‐1^+^CD11b^+^ cells in splenic (SPL) tissue from old and young mice. (E) The cellularity of the SPL is shown. (F) The percent of MΦ (CD11b^+^F4/80^+^) and DC (CD11b^+^CD11c^+^) in the SPL is shown. The results shown represent four young mice and eight naturally aged mice. The data were analyzed using nonparametric two‐way ANOVA (Mann–Whitney) ± SEM (**P < 0.01).

In the SPL, there was a significant increase in the percentage of MDSC in old mice than in young mice (Fig. 3A,B). Similar to the BM, the level of NF‐κB^EGFP^ expressed in splenic MDSC from old mice was greater than that seen in CD11b^+^ or Gr‐1^+^ cells. However, in young mice, the level of expression of NF‐κB^EGFP^ in CD11b^+^ cells appears to be slightly greater than that of MDSC (Fig. 3A,B). A direct comparison between old and young NF‐κB^EGFP^ reporter mice showed that a greater percentage of EGFP^+^ MDSCs from old mice than in young mice (Fig. 3C). The percentage of NF‐κB^EGFP+^ MDSCs was elevated in the SPL of old mice, as well as the overall frequency of MDSCs (Fig. 3D). The cellularity of the spleen was greater in old mice as compared to young (Fig. 3E) which differed from what was seen in the BM. There were no significant differences in the percentage of MΦ or DCs in the spleen (Fig. 3F) or the distribution of MDSC subsets between the age groups (data not shown). These results suggest that EGFP^+^ cells are present at a high frequency in the spleen of old mice.

### Functional analysis of MDSCs

**Figure 4:**
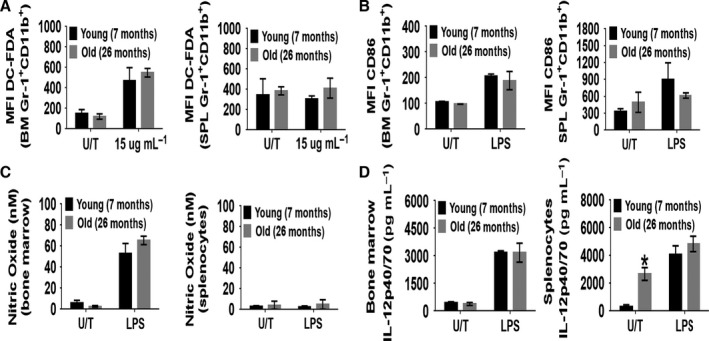
The activation of BM or splenic MDSCs from young and old mice is similar. To test for ROS production by MDSCs, 5 × 10^5^ BM and splenic cells were cultured with 2.5 μm DC‐FDA ± PMA (15 μg mL^−1^) for 30 min. Afterward, the cells were counterstained with anti‐Gr‐1 and anti‐CD11b mAb and analyzed by flow cytometry. (A) The mean fluorescent intensity (MFI) of DC‐FDA in MDSC is shown (U/T = untreated). To determine whether BM or splenic cells could achieve full activation, and their capacity to produce nitric oxide or cytokines, 5 × 10^5^ cells were stimulated with LPS (1 μg mL^−1^) for 24 h. (B) The activation status of MDSCs was assess by measuring the expression of CD86 and shown as the MFI. The supernatants were analyzed for nitric oxide (C) using a Griess reagent‐based kit and cytokine production by ELISA (D). The data shown are from three mice for (A) and (C) while for (B) and (D) the results represent five mice. The data were analyzed using nonparametric two‐way ANOVA (Mann–Whitney) ± SEM (*P < 0.05).

To determine whether the aging process had an impact on MDSC function, we stimulated whole SPL and BM cells with either phorbol 12‐myristate 13‐acetate (PMA) or lipopolysaccharides (LPS) for short time intervals. To screen the production of ROS, cells were incubated with an ROS‐sensitive dye DC‐FDA for 30 min after which the cells were counterstained with Gr‐1 and CD11b mAb and analyzed by flow cytometry. The production of ROS following PMA stimulation did not differ between old and young MDSCs from either the BM or the SPL (Fig. 4A). When BM and splenic cells were stimulated with LPS for 24 h, MDSCs from both old and young mice expressed similar levels of CD86 (Fig. 4B). Supernatants from LPS‐stimulated cells showed that the production of nitric oxide (NO) was also similar between old and young mice (Fig. 4C). We also did not detect differences in the secretion of IL‐12p40/p70 in the supernatants between old and young BM cells following LPS stimulation (Fig. 4D). Naive splenocytes from old mice were producing IL‐12p40/p70, likely indicating a low‐grade inflammatory response associated with aging (Bartlett *et al*., 2012; Tchkonia *et al*., 2013). However, following stimulation with LPS, the production of IL‐12p40/p70 was equalized between the splenocytes from the different aged mice. Taken together, these results suggest that although there are differences in the frequency of MDSC between old and young mice, their MDSCs appear to function similarly.

### MDSCs are increased in the BM of Ercc1^−/∆^ mice and reduced in p65 heterozygous background

**Figure 5:**
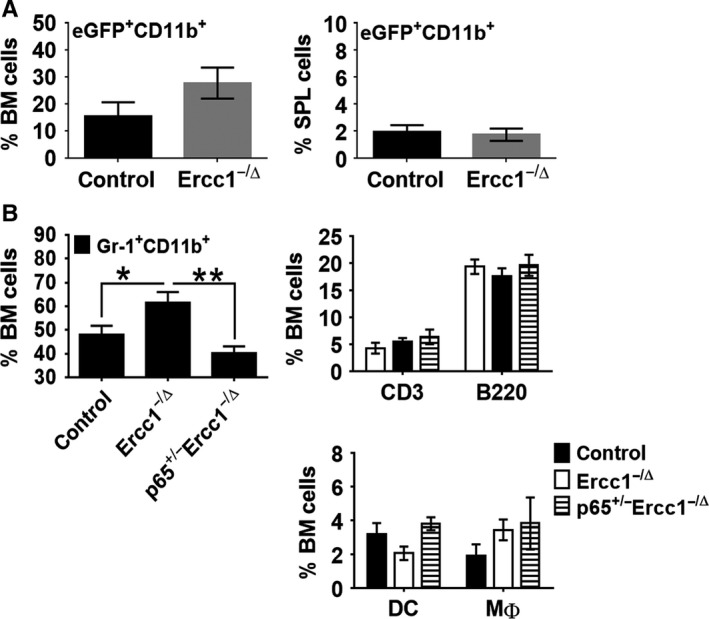
Expansion of myeloid suppressor cells in the BM of the *Ercc1* ^−/∆^ mouse model of a human progeroid syndrome. BM was extracted from the femur and tibia of the hind legs of mice. RBCs were depleted and the BM was washed extensively and prepared for FACS analysis. (A) The percent of NF‐κB^EGFP+^CD11b^+^ cells for both the BM and SPL from *Ercc1* ^−/∆^ NF‐κB^EGFP^ reporter mice (*n* = 5) is shown compared with WT control mice (*n* = 5). (B) The frequency of MDSCs, T cells, and B cells is compared between WT (*n* = 9), *Ercc1* ^−/∆^ (*n* = 6), and p65^+/−^ *Ercc1* ^−/∆^ (*n* = 7) mice. The data were analyzed using nonparametric two‐way ANOVA (Mann–Whitney) ± SEM (*P < 0.05, **P < 0.01).

We hypothesized that if NF‐κB activation and MDSC expansion are age related, these processes should be accelerated in progeroid mice. The BM from *Ercc1* ^−/∆^ mice carrying the NF‐κB^EGFP^ transgene was examined. The *Ercc1* ^−/∆^ mouse has reduced expression of the DNA endonuclease ERCC1‐XPF (Gregg *et al*., 2012; Tilstra *et al*., 2012), which results in accelerated aging in mice, like humans (Niedernhofer *et al*., 2006). Analogous to old NF‐κB^EGFP^ reporter mice, there were more EGFP^+^CD11b^+^ (Fig. 5A) cells in the BM of 3‐month‐old *Ercc1* ^−/∆^ mice compared with control littermates; however, the differences were not statistically significant. No differences in this population of cells were observed in the SPL (Fig. 5A). The percentage of MDSCs was increased in the BM of *Ercc1* ^−/∆^ mice, like old WT mice. Heterozygosity in NF‐κB (p65/RelA), previously shown to improve pathology in *Ercc1* ^−/∆^ mice, resulted in a reduction in the percent of MDSCs back to control levels (Fig. 5B). However, haploinsufficiency of p65 had no effect on the percentages of MΦ, DC, T, and B cells in these mice (Fig. 5B) nor did it improve the cellularity of the BM in these mice (data not shown). In addition, there were no differences in the distribution of the major MDSC subsets in either the BM or SPL (Fig. S2). Collectively, the data suggest that NF‐κB plays a key role in driving the increased percentage of MDSC in progeroid *Ercc1* ^−/∆^ mice like naturally aged mice.

**Figure 6:**
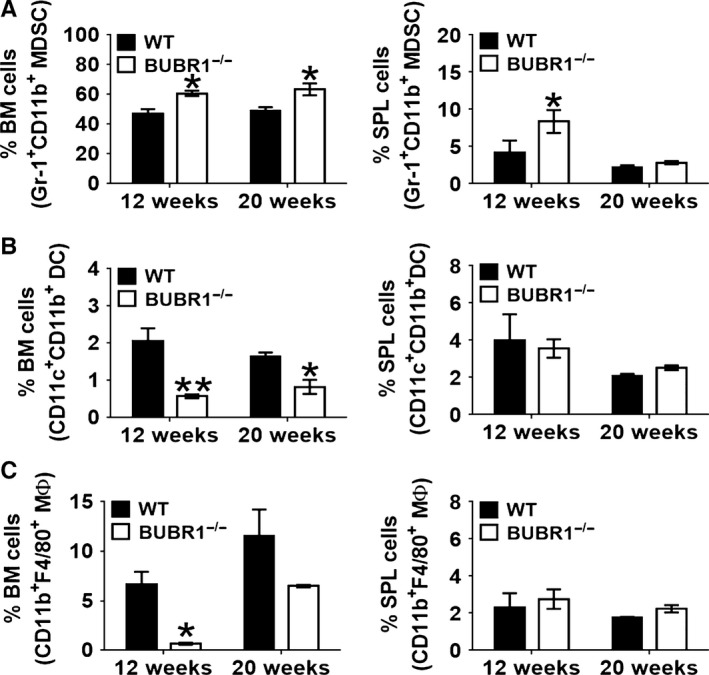
A significant increase in the percent MDSCs detected in the BM and SPL from progeroid Bubr1^H/H^ hypomorphic mice. BM and splenic cells were depleted of RBCs and washed extensively prior to FACS. The percentage of MDSCs and its subsets in the BM (A) and SPL (B) from Bubr1^H/H^ mice is shown in comparison with WT mice. The mice were analyzed at 12 (*n* = 3) and 20 (*n* = 3) weeks of age. The data were analyzed using nonparametric two‐way ANOVA (Mann–Whitney) ± SEM (*P < 0.05, **P < 0.01).

To extend these observations, we analyzed the BM and SPL of a second model of accelerated aging, BubR1 hypomorphic (*BubR1* ^H/H^) mice. BubR1 is a component of the spindle assembly checkpoint that insures orderly progression through mitosis and accurate segregation of chromosomes by contributing to the attachment of the kinetochores to the spindle microtubules (Karess *et al*., 2013). *BubR1* ^H/H^ hypomorphic mice have a short lifespan and exhibit increased senescence and accelerated age‐related decline in muscle, adipose tissue, and the eye (Baker *et al*., 2004, 2008). Similar to our observations from *Ercc1* deficient animals, we observed a significant increase in MDSCs in the BM of *BubR1* ^H/H^ mice (Fig. 6A) at both 12 and 20 weeks. In the SPL, there also was a difference in the percentage of both MΦ and DCs (Fig. 6B). However, in the BM, the percentage of both MΦ and DCs significantly decreased, which was different from what was observed in both naturally aged and *Ercc1* ^−/∆^ mice (Fig. 6C). Of note, the old WT, *Ercc1* ^−/∆^ and *BubR1* ^H/H^ mice were in three different genetic backgrounds (C57Bl/6, C57Bl/6:FVB f1, and C57Bl/6 mixed), indicating that the age‐related changes in immune cell profile is not strain specific.

## Discussion

To examine the regulation of NF‐κB with natural aging, we utilized a transgenic NF‐κB^EGFP^ reporter mouse. An increase in the percent of cells positive for the NF‐κB^EGFP^ reporter was observed in the pancreas, gastrointestinal tract, and liver with age (Tilstra *et al*., 2012; data not shown). This suggests that stochastic events that occur over time can lead to activation of NF‐κB. Here, we demonstrate that the greatest NF‐κB^EGFP^ signal in the BM was from Gr‐1^+^CD11b^+^ MDSCs, with a fraction of EGFP^+^ MDSCs increasing with the age of mice. We also detected a smaller, but still significant increase in the NF‐κB^EGFP^ signal in splenic MDSCs. There was also an overall increase in the percent of MDSCs, both EGFP positive and negative with aging in both tissues. Notably, when aging is accelerated, as in progeroid mouse strains, the accumulation of NF‐κB^EGFP+^ MDSCs is accelerated, illustrating that it is an age‐related event. Also of note, despite the increase in NF‐κB activity and the overall percent of MDSCs, the aged MDSCs appeared to function similarly to those from young mice. It is likely that activation of NF‐κB is mediated by cellular stress including oxidative or genotoxic stress or inflammation, which then drives expansion of the MDSCs. Indeed, genotoxic or oxidative stress is known to increase nuclear ATM activity, which phosphorylates the IKK kinase regulatory subunit NEMO to activate the NF‐κB pathway (McCool & Miyamoto, 2012; Salminen *et al*., 2012). It is also possible that inflammatory factors secreted by senescent cells accumulating with age can drive NF‐κB activation.

Interestingly, reduction in the level of NF‐κB/RelA prevents the expansion of MDSC in progeroid *Ercc1* ^−/∆^ mice. This suggests that activation of NF‐κB with age drives, at least in part, expansion of MDSCs. This also suggests that although not all the MDSCs are positive for EGFP, NF‐κB activity is increased in this cell population. How NF‐κB is activated in a subset of immune cells is unclear. Previous studies have shown that cytokines like TNF‐α that stimulate NF‐κB can drive the expansion of MDSC (Sade‐Feldman *et al*., 2013). TNF‐α also can block the differentiation of immature myeloid cells into MΦ/DCs, driving differentiation only to the MDSC stage, as well as contribute to the suppressive functional activity of MDSC. The *Ercc1* ^−/∆^ mice, like naturally aged mice, have an increase in TNF‐α (Chen *et al*., 2013). However, it is likely that NF‐κB also is activated by stochastic cell autonomous events such as cellular stress including genomic and oxidative stress with age.

MDSCs represent a group of myeloid cells that originate in the BM possessing a variety of suppressive actions already described (Gabrilovich & Nagaraj, 2009; Ribechini *et al*., 2010; Talmadge & Gabrilovich, 2013). MDSCs suppress lymphocytes through classical mechanisms involving cytokines like IL‐10. However, MDSC‐mediated suppression is most effective on a cell‐to‐cell level, either through direct receptor–ligand interaction or through small short‐lived chemical mediators (Gabrilovich & Nagaraj, 2009; Talmadge & Gabrilovich, 2013). MDSC production of small short‐lived chemical mediators is associated with the metabolism of L‐arginine (Rodriguez & Ochoa, 2008). MDSCs express the enzymes arginase I and iNOS that metabolize L‐arginine into either urea and L‐ornithine or nitric oxide, respectively. The expression of these enzymes can be influenced by the type of differentiated T cells they encounter. For instance, Th1 cells (IFNγ^+^) can drive MΦ to express iNOS (Hesse *et al*., 2001; Ribechini *et al*., 2010). However, we observed no difference in the ability of BM or splenic cells from old mice to produce NO following stimulation with LPS compared with young mice (Fig. 4C). Furthermore, when we assess the activation of MDSC (CD86 MFI), no differences were observed between old and young MDSC (Fig. 4C) or between WT and *Ercc1* ^−/∆^ mice (data not shown).

MDSCs can modulate the function of T cells through an oxidative stress mechanism involving the production of ROS. ROS, a by‐product of NADPH oxidase, is produced by MDSCs in response to certain cytokines like IFNγ, IL‐6, or TGF‐β (Gabrilovich & Nagaraj, 2009). ROS can suppress T‐cell secretion of cytokines and induce apoptosis (Malmberg *et al*., 2001). However, in this study, we observed no difference in the production of ROS by MDSCs from either the BM or SPL between old and young mice (Fig. 4A). This functional similarity between MDSCs derived from mice of different ages extends to their expression of IL‐10 (data not shown), and IL‐12 (Fig. 4D), where no significant differences were detected. The one exception was the increased production of IL‐12 by unstimulated splenocytes from old mice. This production of IL‐12 may reflect the ongoing low‐grade inflammatory response present in geriatric humans and aged mice, independent of infection, which is commonly referred to ‘inflammaging’ (Bartlett *et al*., 2012; Tchkonia *et al*., 2013).

MDSCs can also exhibit pro‐inflammatory activity. For example, MDSCs can exacerbate the immune response in mouse models of experimental autoimmune encephalomyelitis and collagen‐induced arthritis (Yi *et al*., 2012; Guo *et al*., 2014; Zhang *et al*., 2015). In these studies, MDSCs enhanced the generation of Th17 cells through a mechanism dependent on IL‐1β. *In vitro*, the production of IL‐17A is enhanced in the presence of MDSCs and decreased by an IL‐1 inhibitor (Yi *et al*., 2012; Zhang *et al*., 2015). Thus, the increase in MDSCs with aging may contribute to inflammaging.

The majority of our understanding of MDSC biology comes from the role these cells play in tumor pathobiology. MDSCs are detected in tumors from cancer patients and from tumor‐bearing mice. Furthermore, MDSCs are present in the blood of cancer patients, while in tumor‐bearing mice, the percentage of MDSC in the spleen can reach as high as 30% (Diaz‐Montero *et al*., 2009; Hurez *et al*., 2012; Jackaman *et al*., 2013; Verschoor *et al*., 2013). Disproportionate expansion of MDSCs can impede in the migration of T cells. Tumor‐derived factors can recruit and induce the differentiation of immature myeloid cells into MDSCs. Cytokines like granulocyte–macrophage‐derived soluble factor (GM‐CSF) and vascular endothelial growth cell factor (VEGF), which promote myelopoiesis toward MDSCs, are factors secreted by tumor cells (Ostrand‐Rosenberg & Sinha, 2009; Youn & Gabrilovich, 2010; Gabrilovich *et al*., 2012; Talmadge & Gabrilovich, 2013). Our data shows not only an expansion of MDSCs in the BM, but also a preferential expansion of MΦ over DC (Fig. 2F) with aging. This suggests that the differentiation of myeloid cells to DCs is actively suppressed. Tumor‐derived MDSCs also suppress T‐cell activity by facilitating the recruitment and induction of Tregs (Huang *et al*., 2006; Hurez *et al*., 2012). The induction of Tregs associated with the expansion of MDSC could be exacerbated and thus more prominent during tumor development (Rosin *et al*., 2011). However, both natural aged and progeroid Ercc1^−/∆^ mice show no differences in Tregs when compared to appropriate control mice (data not shown). Thus, the mechanisms that MDSCs use to mediate immune suppression apparently are intact in aged mice.

A reduction in the ability to combat infectious diseases and tumor immune surveillance are comorbidities associated with old age. Studies have shown an increase in the frequency of the MDSC population in both elderly patients and mice (Hurez *et al*., 2012; Jackaman *et al*., 2013; Verschoor *et al*., 2013). In one particular study, elderly patients were shown to have elevated levels of CD33^+^HLA‐DR^−^CD11b^+^CD15^+^ MDSCs circulating in their blood compared with healthy adults (Verschoor *et al*., 2013). In frail elderly patients and those with a history of cancer, the frequency of MDSCs is further elevated. In naturally aged mice, the frequency of MDSCs in the SPL, lymph nodes, and BM is elevated compared with young mice, similar to our current findings (Enioutina *et al*., 2011; Hurez *et al*., 2012; Jackaman *et al*., 2013). However, here we extend this analysis by demonstrating that the increase in the percentage of MDSCs with age is mediated by NF‐κB/RelA activation in not only naturally aged mice, but also two different progeriod mouse strains. Also, within the multiple cell populations in the bone marrow and spleen, NF‐κB/RelA is activated with age primarily in MDSCs.

Although it has been demonstrated that the percent of MDSC as well as MDSC with NF‐κB activation increases with accelerated and natural aging, the role of the MDSC, if any, in the aging process is unclear. It is also unclear whether the expansion of MDSCs drives aging or only is a consequence of aging. However, given that MDSCs accumulate in the spleen, peripheral lymph nodes, bone marrow, and blood of normal aged mice and are significantly increased in the circulation of aged individuals, it is likely that MDSCs contribute to age‐associated immune dysfunction. Similarly, the increase in MDSCs with aging could contribute to the increased risk of cancer.

## Experimental procedures

### Mice

Naturally aged C57BL/6 wild‐type (WT) mice were purchased for the National Institute of Aging and maintained under specific pathogen‐free conditions. The NF‐κB^EGFP^ reporter mice (C57BL/6) were provided by Christian Jobin (UNC, Chapel Hill) and have been described previously (Magness *et al*., 2004). Additionally, *Ercc1* ^−/∆^ and Bubr1^H/H^ mice were housed and maintained under specific pathogen‐free conditions and experimented with procedures approved by the Institutional Animal Care and Use Committee of the University of Pittsburgh and Scripps Florida (Baker *et al*., 2004, 2008; Niedernhofer *et al*., 2006). All mice used in this study were euthanized by carbon dioxide fixation.

### Phenotypic analysis of BM and spleen by flow cytometry

The ends of bones were clipped and the BM flushed with a syringe prefilled with media using a 27‐gauge needle. The BM was converted into single cell suspension by mechanically disrupting the small aggregates of tissue by flushing it through an 18‐gauge needle. The BM was washed with sterile PBS and then red blood cells (RBC) were depleted using RBC lysis buffer (150 mm ammonia chloride, 1 mm sodium bicarbonate, and 0.1 mm EDTA at pH 7.7). The cells were extensively washed and passed through a cell strainer. Subsequently, the cells were resuspended in FACS buffer (2% FBS, 1× PBS, 2 mm EDTA, and 0.04% sodium azide) at a concentration of 3.75 × 10^6^ cells per ml. A 200‐μL aliquot of each sample was transferred into 96‐well polypropylene round‐bottom plates (BD Bioscience). Fc receptors were blocked using anti‐CD16/CD32 mAb at a 1:600 dilution for 20 min at 10°C. The cells were stained with fluorochrome‐conjugated mAb at the appropriate titer for 45 min at 10°C (anti‐CD11b, CD86, CD4, and Fc block were purchased from BD Pharmingen; anti‐CD19, B220, Ly‐6G, Ly‐6C, F4/80, Gr‐1, and CD11c mAb were purchased from eBioscience). The cells were washed with FACS buffer twice and then fixed in 2% paraformaldehyde. For each sample, 50 000 cells were passed through a BD LSR II flow cytometer (BD Bioscience) and analyzed using Flowjo (Tristar, Inc. Ashland, OR).

### *In vitro* functional analysis of BM MDSC

BM cells from three naturally aged mice were pooled, the RBC depleted and cells washed thoroughly with sterile PBS. The BM cells were resuspended at 5 × 10^5^ cells per well and stimulate with LPS (1 μg mL^−1^) for 24 h. The culture supernatants were saved and analyzed for the presence of nitric oxide (NO) utilizing a NO kit from Cayman Chemical (Ann Arbor, MI USA). Additionally, the treated cells were phenotypically analyzed for the expression of activation markers by flow cytometry as described in the previous subsection. To assay for ROS production, 5 × 10^5^ cells were cultured with 2.5 μm oxidative sensitive dye DC‐FDA (Invitrogen) and PMA (30 μg mL^−1^) at 37°C for 30 min. The cells then were counterstained with anti‐Gr‐1 and anti‐CD11b mAb and analyzed by flow cytometry. As a comparison, splenocytes were tested in parallel with the BM cells.

## Author's contributions

RRF designed and performed experiments and wrote the manuscript. CLC, JC, and, SJM performed experiments while BCL, DJB, LJN, and PDR helped to design experiments and edited this manuscript.

## Funding

This work was supported by grants AG024827, AR051456, and AG043376 from the National Institutes of Health. R.R.F was supported by a T32 grant from NIH on Autoimmunity and Immunopathology.

## Conflict of interest

The authors report no conflict of interest relevant to this article.

## Supporting information


**Fig. S1** A majority of bone marrow NF‐κB^EGFP+^ cells are Gr‐1^+^CD11b^+^ MDSC but not in the spleen of naturally aged mice.

**Fig. S2** No difference in the percentage of MDSC subsets in the bone marrow of naturally aged or the *Ercc1* ^−/∆^ progeroid mouse model of aging.

**Fig. S3** A greater level of NF‐κB activity detected in MΦ and DCs in the BM of old mice compared to young adult.

**Fig. S4** The expression of CD86 is increased in EGFP^+^ MΦ and DCs in the BM of old mice.
