# Supplementary figures and images for: Expansion of myeloid‐derived suppressor cells with aging in the bone marrow of mice through a NF‐κB‐dependent mechanism
Source: Aging Cell. 2017 Feb 23;16(3):480–7. doi: 10.1111/acel.12571 (PMC5418207; doi:10.1111/acel.12571)

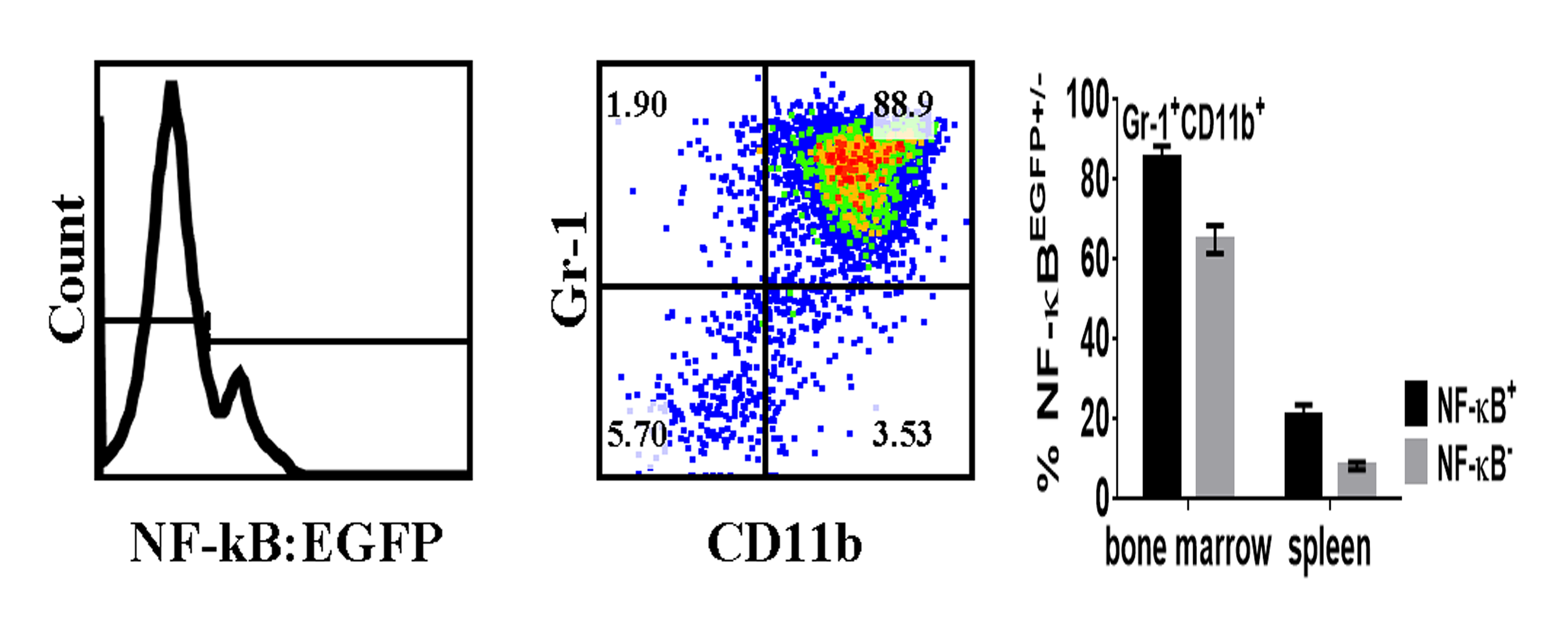

Supplement: Supplementary file 1 — Fig. S1 A majority of bone marrow NF‐κBEGFP+ cells are Gr‐1+CD11b+ MDSC but not in the spleen of naturally aged mice. [file ACEL-16-480-s001.tif]

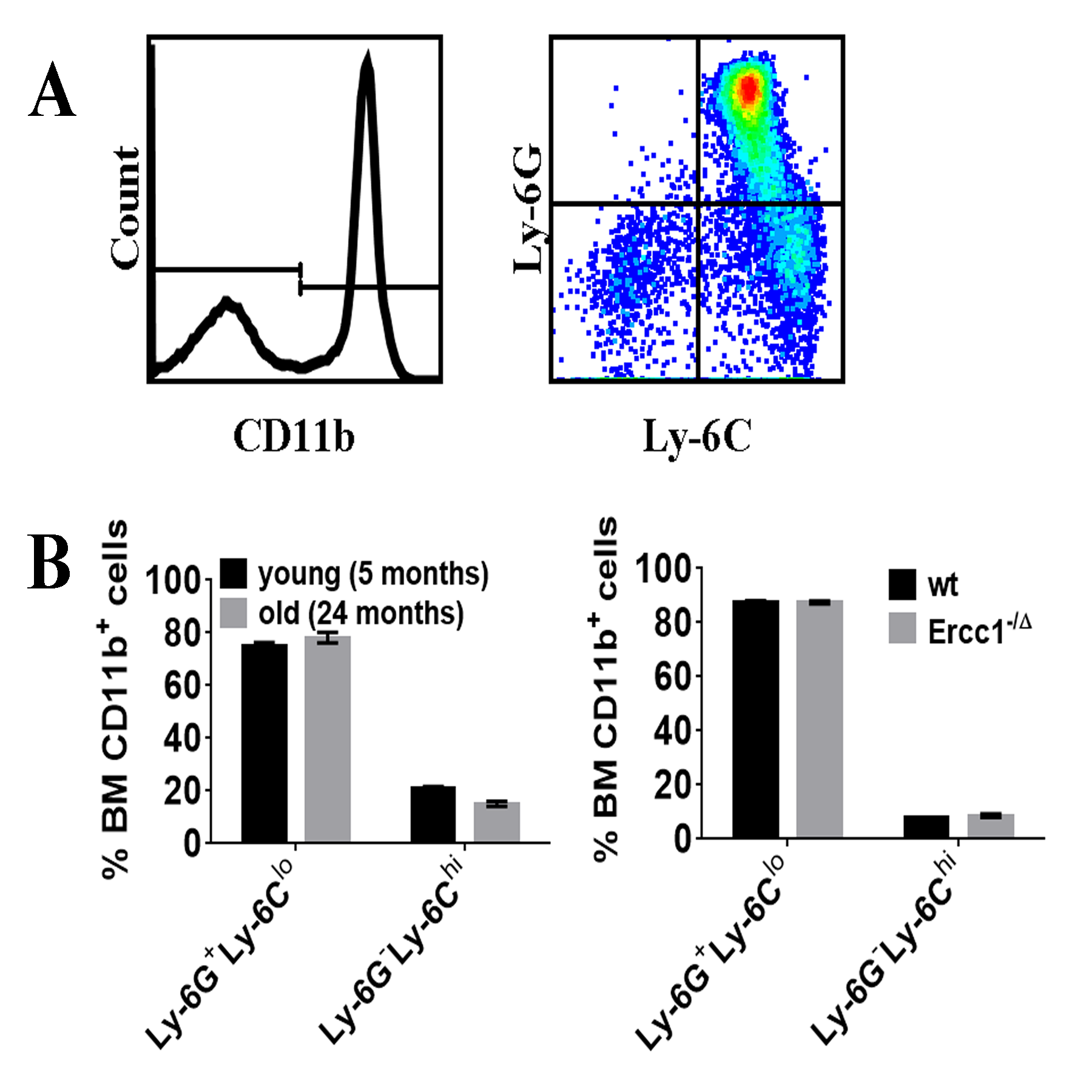

Supplement: Supplementary file 2 — Fig. S2 No difference in the percentage of MDSC subsets in the bone marrow of naturally aged or the Ercc1 −/∆ progeroid mouse model of aging. [file ACEL-16-480-s002.tif]

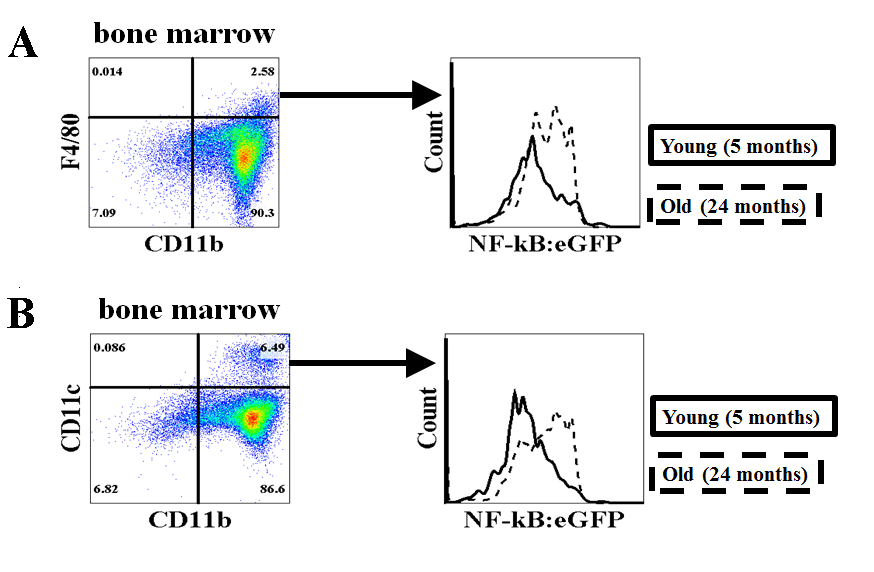

Supplement: Supplementary file 3 — Fig. S3 A greater level of NF‐κB activity detected in MΦ and DCs in the BM of old mice compared to young adult. [file ACEL-16-480-s003.tif]

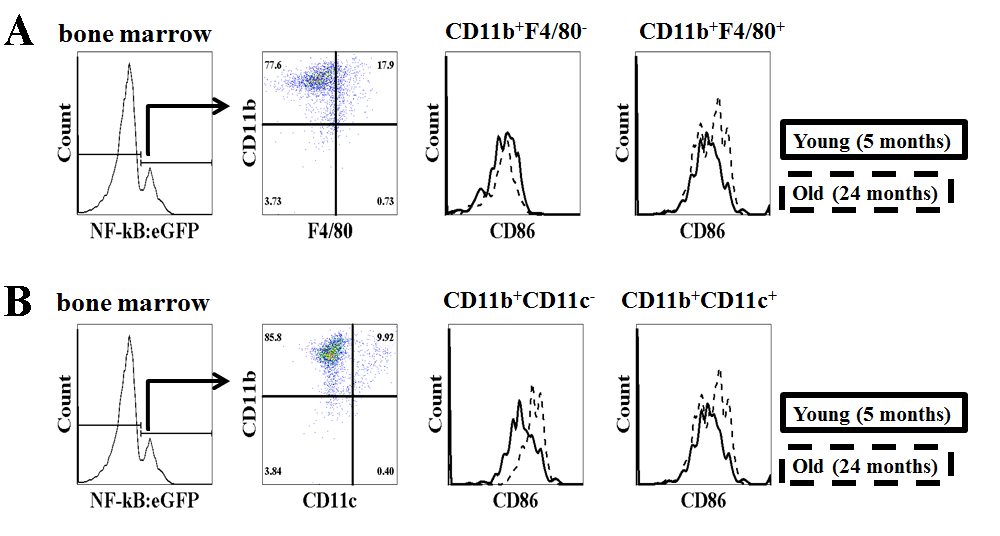

Supplement: Supplementary file 4 — Fig. S4 The expression of CD86 is increased in EGFP+ MΦ and DCs in the BM of old mice. [file ACEL-16-480-s004.tif]
